# Macrophage κ-opioid receptor inhibits hypoxic pulmonary hypertension progression and right heart dysfunction via an SCD1-dependent anti-inflammatory response

**DOI:** 10.1016/j.gendis.2025.101604

**Published:** 2025-03-18

**Authors:** Qiaojuan Wang, Jiayuan Liu, Renqi Li, Sihan Kong, Yinjie Wang, Guoyang Huang, Shumiao Zhang, Na Feng, Xiaoming Gu, Yali Liu, Ming Jia, Feng Fu, Jun Li, Juan Li, Jianming Pei

**Affiliations:** a Department of Physiology and Pathophysiology, National Key Discipline of Cell Biology, Fourth Military Medical University, No. 169 Changle West Road, Xi'an, Shaanxi 710032, China; b Department of Cardiology, Xijing Hospital, Fourth Military Medical University, Xi'an, Shaanxi 710032, China

**Keywords:** κ-opioid receptor, Hypoxic pulmonary hypertension, Inflammation, Macrophage, SCD1

## Abstract

We aimed to investigate the effects and mechanism(s) of macrophage κ-opioid receptor (κ-OR) on macrophage inflammatory response and hypoxic pulmonary hypertension (HPH). Macrophage κ-OR-deficient mice (κ-OR^ΔMac^) and their wild-type control mice (κ-OR^fl/fl^) were subjected to HPH or control groups. Mice with HPH presented significantly decreased expression of κ-OR in peritoneal macrophages. Compared with the κ-OR^fl/fl^ + control group, the κ-OR^fl/fl^ + HPH group presented increased right ventricular pressure, pulmonary vascular remodeling, and right ventricular hypertrophy and dysfunction; infiltration of M1 macrophages around pulmonary vessels; increased NLRP3 protein expression; and the release of the inflammatory cytokines. Macrophage κ-OR deficiency significantly aggravated the phenomenon mentioned above. At the cellular level, macrophages with κ-OR deficiency also aggravated lipopolysaccharide-induced inflammation. In addition, administering the κ-OR-selective agonist U50,488H significantly inhibited the inflammatory response in macrophages. The co-culture experiments revealed that U50,488H-treated macrophages inhibited the proliferation of pulmonary artery smooth muscle cells. Furthermore, our RNA sequencing and western blotting results revealed that κ-OR increases stearoyl coenzyme desaturase 1 (SCD1) expression in macrophages. Macrophage κ-OR knockdown significantly decreased SCD1 expression both in the lung tissues of HPH mice and in cultured macrophages. Moreover, SCD1 overexpression significantly suppressed the inflammatory response in lipopolysaccharide-treated macrophages, whereas the pharmacological inhibition of SCD1 increased the response. These results demonstrated that macrophage κ-OR inhibited HPH and right heart dysfunction by up-regulating SCD1, which inhibited macrophage inflammatory responses and pulmonary artery smooth muscle cell proliferation. This study provides more evidence to support the potential therapeutic role of κ-OR activation in the treatment of HPH.

## Introduction

Pulmonary hypertension (PH) is a pathological condition characterized by elevated pulmonary artery pressure, increased pulmonary vascular resistance, and pulmonary vasculature remodeling, ultimately leading to right ventricular failure. The reduced pulmonary blood flow, increased right heart strain, and functional deterioration in PH patients pose significant threats to human life and well-being worldwide.^1^^,^^2^ PH is classified into five categories,^3^ with hypoxic pulmonary hypertension (HPH), also known as hypoxia-induced PH, comprising the third category. HPH represents a complex disorder that has remained enigmatic despite extensive research efforts over several decades, and it remains an interesting and challenging issue in the field.^4^ HPH is characterized by sustained vasoconstriction and vascular remodeling.^5^ Vasoconstriction and relaxation are mediated by a variety of vascular endothelial substances, such as endothelin-1, prostaglandin, and nitric oxide.^6^ Furthermore, the excessive proliferation of pulmonary artery smooth muscle cells (PASMCs) contributes to vascular remodeling.^7^, ^8^, ^9^

Various factors interact and collaborate to affect pulmonary vessels, ultimately leading to irreversible vascular remodeling.^10^ Moreover, the inflammatory response plays a crucial role in HPH occurrence and progression, thus representing a hot topic of investigation in this field. Multiple immune cells, including neutrophils, macrophages, dendritic cells, mast cells, T lymphocytes, and B lymphocytes, characteristically accumulate around the pulmonary artery during pulmonary vascular remodeling.^11^ Perivascular inflammatory infiltration represents a notable pathological characteristic among most PH patients. Additionally, elevated levels of cytokines, chemokines, and inflammatory factors are detected in individuals with PH.^12^^,^^13^ Therefore, pulmonary inflammation is recognized as a contributing factor to the development of pulmonary hypertension.^14^ The study of pulmonary inflammation is highly important for HPH treatment.

Among the multiple types of immune cells, macrophages are the predominant inflammatory cell type infiltrating PH lesions. A variety of studies in experimental animal models have shown that perivascular macrophages play a central role in the vascular remodeling associated with PH.^15^, ^16^, ^17^ During the early period of hypoxic exposure, macrophages accumulate around pulmonary vessels, exhibit a hypoxic response, and release proinflammatory cytokines. Subsequently, the perivascular accumulation of macrophages decreases, resulting in tissue repair and anti-inflammatory programming states.^18^ Macrophages in the lung are heterogeneous cell populations that occupy different niches and exhibit microenvironment-specific phenotypes and functions. M1-type macrophages amplify inflammation by secreting proinflammatory factors, whereas M2-type macrophages promote tissue repair and play a major role in pulmonary vascular remodeling.^19^ More importantly, the balanced polarization of M1/M2 macrophages governs the fate of an organ during inflammation or injury. Inhibiting the release of M1 macrophage-derived proinflammatory cytokines effectively protects against experimental PH progression and ameliorates the right ventricular burden in hypoxic mouse models.^15^^,^^20^ A recent study reported that treatment with immunoregulatory macrophages (M2_regs_) reduces alveolar macrophage inflammation and attenuates right ventricular hypertrophy, right ventricular systolic pressure, and vascular remodeling in hypoxic mice.^21^

The κ-opioid receptor (κ-OR) is a prominent subtype of opioid receptor (OR) that has been identified in both the central nervous system and peripheral tissues.^22^ Compared with other ORs,^23^ κ-OR plays a pivotal role in peripheral blood vessels and has also been detected in pulmonary arteries. Our previous studies demonstrated that κ-OR activation by the exogenous agonist U50,488H effectively prevents the development of HPH.^24^^,^^25^ κ-OR activation effectively inhibits HPH-induced lung inflammation, but the underlying mechanisms are largely unknown.^26^^,^^27^ The present study aims to investigate the regulatory effect of macrophage κ-OR on pulmonary inflammation in HPH and the underlying mechanism.

## Materials and methods

### Animals

Lyz2-Cre mice (Shanghai Model Organisms Center, China) were bred with κ-OR-floxed mice (Shanghai Model Organisms Center) to generate mice with myeloid-specific deletion of κ-OR (κ-OR^ΔMac^) and the corresponding κ-OR^fl/fl^ littermate controls. κ-OR^fl/fl^ and κ-OR^ΔMac^ mice were housed in accordance with animal care standards at the Experimental Animal Center of Air Force Military Medical University (in line with animal feeding standards). Specific pathogen-free C57BL/6J mice, 6–8 weeks old and weighing 200 ± 20 g, were obtained from the same center. The animal experiment was conducted following the approval of the Experimental Animal Care and Use Committee of Air Force Military Medical University and adhered to the National Institutes of Health Guidelines for the Care and Use of Laboratory Animals (eighth edition).

### Mouse model of HPH

A mouse model of HPH was established as previously reported.^28^ The mice in the hypoxia group were exposed to a hypoxic environment (Shanghai Tow Intelligent Technology, China) at an altitude of 5000 m (50 kPa) with a gas mixture containing 10% oxygen for 23 h per day over 4 weeks. The mice in the hypoxia group also received weekly subcutaneous injections of SU5416 (S8442; Sigma, USA) for 1–4 weeks. The mice in the control group were maintained under normal oxygen and pressure conditions. Sodium lime was used within the chamber to absorb CO_2_ and water vapor, whereas an ice bag was utilized for cooling if necessary due to the elevated temperature inside the chamber.

### Measurement of cardiac function

Echocardiography was performed on anesthetized animals at the indicated time points using a VEVO 2100 ultrasound system (Visual Sonics, Canada). The animals were anesthetized with 2.5% isoflurane for evaluation. M-mode images of right ventricular wall thickness, tricuspid annular plane systolic excursion, left ventricular end-systolic diameter, and left ventricular end-diastolic diameter values were obtained.

### Measurement of right ventricular pressure and mean pulmonary arterial pressure

After hypoxia + SU5416 treatment for 4 weeks, the mice were anesthetized with pentobarbital sodium (60 mg/kg, intraperitoneally) and placed in the supine position. The right ventricular systolic pressure was measured using a micropressure sensor (Chengdu Instrument, China). Briefly, a scalp needle was rapidly inserted into the third and fourth intercostal spaces to access the right ventricle, and 10 data points were continuously recorded and analyzed using the Lab Chart software RVS Biosignal acquisition and analysis system. The right ventricular hypertrophy index, also known as the Fulton index [right ventricular/(left ventricular + septum)], was calculated. This anatomical measurement involved calculating the ratio of the right ventricle weight to the combined weight of the left ventricle plus the septum in control and hypoxia-exposed mouse hearts.

### Histological analysis

The corresponding tissue sections were prepared according to the standard experimental procedures of Servicebio (China), including pathological tissue sampling and fixation, embedding, paraffin sectioning, freezing, and other experimental procedures. The paraffin sections were immersed sequentially in environmentally friendly dewaxing transparent liquid I for 20 min, environmentally friendly dewaxing transparent liquid II for 20 min, anhydrous ethanol I for 5 min, anhydrous ethanol II for 5 min, and 75% ethyl alcohol for 5 min. Then, samples were rinsed with tap water. The frozen sections were removed from the −20 °C refrigerator and stored at room temperature, fixed with tissue fixation solution for 15 min, and then rinsed with running water. The sections were treated with high-definition constant staining pretreatment solution for 1 min. The sections were placed in hematoxylin solution for 3–5 min and rinsed with tap water. Then, the sections were treated with a hematoxylin differentiation solution and rinsed with tap water. The sections were treated with a hematoxylin bluing solution and rinsed with tap water. The sections were placed in 95% ethanol for 1 min and eosin dye for 15 s. The sections were placed in absolute ethanol I for 2 min, absolute ethanol II for 2 min, absolute ethanol III for 2 min, normal butanol I for 2 min, normal butanol II for 2 min, xylene I for 2 min, and xylene II for 2 min, after which they were sealed with neutral gum. The sealed sections were then subjected to microscope inspection, image acquisition, and analysis.

### Immunofluorescence staining

The slides were deparaffinized and subjected to antigen retrieval in a hot citric acid buffer. Under room temperature conditions, the slides were permeabilized with 0.2% Triton-100 for 10 min and blocked with 1% bovine serum albumin in phosphate-buffered saline solution for 1 h. The slides were then incubated overnight with one of the following primary antibodies: alpha-smooth muscle actin (α-SMA, GB13044, Servicebio, 1:600), F4/80 (GB113373, Servicebio, 1:5000), inducible nitric oxide synthase (iNOS, GB11119, Servicebio, 1:5000), or cluster of differentiation CD206 (GB113497, Servicebio, 1:10,000) at 4 °C. Primary antibodies were visualized with a goat anti-rabbit IgG (H + L) secondary antibody conjugated with Alexa Fluor 488 (GB25303, Servicebio, China) or a goat anti-rabbit IgG (H + L) secondary antibody conjugated with CY3 (GB21303, Servicebio, China). Nuclei were stained with 4′,6-diamidino-178 2-phenylindole (DAPI, GB1012, Servicebio). Images were acquired with a Nikon Eclipse E100 microscope and a Nikon DS-U3 camera.

### Macrophage studies

Peritoneal macrophages were isolated from mice following our previously established methods.^29^ Three days before the experiment, the mice were injected with 3% sodium thioglycolate (Becton, Dickinson and Company, USA) at a dose of 300 mL/mouse. On the day of the experiment, the mice were euthanized by cervical dislocation. The mice were disinfected by immersion in 75% alcohol for 5 min (the alcohol beaker was placed on ice during this step) and then transferred to a clean, sterile workbench. The abdominal skin and muscles of the mice were incised using scissors, and the muscles on both sides were clamped with hemostatic forceps. Precooled phosphate-buffered saline solution was dripped into the abdomen using a sterile dripper, and the phosphate-buffered saline solution collected from the abdominal cavity was transferred to a 15-mL centrifuge tube. This process was repeated several times until a volume of 12–15 mL was reached. The cell suspension was centrifuged at 1200 rpm for 5 min, followed by resuspension in medium containing 10% fetal bovine serum. The cell culture plates were incubated at 37 °C in a CO_2_ incubator for 4–8 h and then washed twice with phosphate-buffered saline to remove unattached cells. Peritoneal macrophage purity was detected by flow cytometry. PE-conjugated anti-mouse F4/80, FITC-conjugated anti-mouse CD11b, and their corresponding isotype control antibodies were added for detection and analysis.

Peritoneal macrophages were transfected with adenovirus carrying EGFP (AD-EGFP) or adenovirus carrying EGFP and Cre recombinase (AD-Cre) (HANBIO Biotechnology, China). The adenovirus titer used in this study was 1.2 × 10^10^ PFU/mL. As previously mentioned,^30^ the infection was performed at a multiplicity of infection of 20. After 48 h of adenovirus transfection, the fluorescence expression of GFP was detected, and the infection efficiency reached more than 80%. These cells were utilized in subsequent experiments.

The lentiviral vector encoding CMV-SCD1-3flag-EF1 and the negative control were constructed by HANBIO Biotechnology (China). RAW26.7 cells were purchased from Procell Life Science & Technology (China). When the cells reached 80% confluence, they were centrifuged at 1000 *g* for 5 min. The cells were collected and suspended in 2 mL of high-glucose Dulbecco's modified Eagle medium containing 15% fetal bovine serum and then cultured at 37 °C in 5% CO_2_. RAW264.7 cells were transfected with lentivirus (pHBLV-CMV-SCD1-3flag-EF1-Zsgreen-T2A-PURO) and then screened and maintained with 4 μg/mL puromycin. The stable RAW264.7 strain was used for subsequent experiments.

### ELISA

ELISA kits (Signalway Antibody, USA) were used for interleukin 6 (IL-6, EK0499), interleukin-1beta (IL-1β, EK0502), and Tumor necrosis factor-alpha (TNF-α, EK0497) protein detection in the culture medium. Initially, the culture supernatant sample was chilled on ice while the ELISA plate was allowed to equilibrate at room temperature for 10 min. Subsequently, 100 μL of standard or sample was added to each well of the enzyme plate, 100 μL of sample mixture was added to the blank well, and 50 μL of enzyme mixture was added to each well (excluding the blank well). The plate was then incubated at 37 °C for 120 min, followed by liquid removal and three washes per well. Next, 100 μL of enzyme coupling working mixture was added to each well and incubated at 37 °C for an additional 30 min. After the liquid was discarded and five subsequent washes were performed per well, 100 μL of substrate mixture was added to each well and incubated in the dark at 37 °C for 20 min. Finally, 100 μL of termination mixture was added to stop the reaction. The absorbance at a wavelength of 450 nm was measured for each well to calculate the sample concentration using a standard curve.

### RNA sequencing analysis

Differential gene expression analysis using RNA sequencing was performed by Novogene Company (China). Total RNA was extracted from macrophages with TRIzol (Invitrogen, USA) according to the manufacturer's instructions. Total RNA was used as input material for the RNA sample preparations. The data were subsequently converted into sequencing libraries using a KAPA Stranded RNA-Seq Library Prep Kit (Illumina, USA). Sequencing was performed with an Illumina HiSeq 4000 platform after RNA library quality was assessed using an Agilent 2100 Bioanalyzer (Agilent Technologies, USA). |log_2_fold change| ≥ 1 and *P*_adj_ ≤ 0.05 were used to classify the differentially expressed genes. Pathway analysis was performed using the Kyoto Encyclopedia of Genes and Genomes (KEGG) database using up-regulated or down-regulated differentially expressed genes.

### Cell migration and invasion assays

Cell coculture was performed using transwell in 24-well plates (pore sizes of 0.4 μm; Merck, USA). RAW264.7 macrophages were seeded in the upper compartment, and PASMCs were seeded in the lower compartment. Dulbecco's modified Eagle medium (Gibco, USA) containing 10% fetal bovine serum (Gibco) was added. The transwell system was incubated at 37 °C in a humid atmosphere with 5% CO_2_.

### Quantitative real-time PCR

The relative IL-1β, IL-6, TNF-α, CD86, and CD206 mRNA expression levels were detected using quantitative real-time PCR. Total RNA was isolated from lung tissue and macrophages using TRIzol reagent (15596018CN, Thermo Fisher Scientific, USA), and 1 mg of RNA was then reverse-transcribed using a reverse transcription kit (AG11728, Accurate Biology). PCR was performed on a Bio-Rad real-time PCR system. Each reaction consisted of a DNA template along with forward and reverse primers combined with Power SYBR Green PCR master mixture. The mRNA levels were normalized to those of β-actin using the 2^−ΔΔCt^ method. The primers used in this study are presented in Table 1.

**Table 1:** Real-time PCR primers for mice.

Genes	Forward primer (5′—3′)	Reverse primer (5′—3′)
Cre	TGGCATTTCTGGGGATTGCT	AACCAGCGTTTTCGTTCTGC
Oprk (κ-OR)	GAATCCGACAGTAATGGCAGTG	GACAGCGGTGATGATAACAGG
IL-6	TAGTCCTTCCTACCCCAATTTCC	TTGGTCCTTAGCCACTCCTTC
IL-1β	GAAATGCCACCTTTTGACAGTG	TGGATGCTCTCATCAGGACAG
β-actin	AACAGTCCGCCTAGAAGCAC	CGTTGACATCCGTAAAGACC
Scd2	GCATTTGGGAGCCTTGTACG	AGCCGTGCCTTGTATGTTCTG
Scd1	TTCTTGCGATACACTCTGGTGC	CGGGATTGAATGTTCTTGTCGT
Acat2	CCCGTGGTCATCGTCTCAG	GGACAGGGCACCATTGAAGG
Fads2	AAGGGAGGTAACCAGGGAGAG	CCGCTGGGACCATTTGGTAA
Acsl3	AACCACGTATCTTCAACACCATC	AGTCCGGTTTGGAACTGACAG
Elovl6	GAAAAGCAGTTCAACGAGAACG	AGATGCCGACCACCAAAGATA
Acot1	ATACCCCCTGTGACTATCCTGA	CAAACACTCACTACCCAACTGT
Cyp51	GACAGGAGGCAACTTGCTTTC	GTGGACTTTTCGCTCCAGC
Dhcr24	CTCTGGGTGCGAGTGAAGG	TTCCCGGACCTGTTTCTGGAT
Fdft1	ATGGAGTTCGTCAAGTGTCTAGG	CGTGCCGTATGTCCCCATC
Msmo1	AAACAAAAGTGTTGGCGTGTTC	AAGCATTCTTAAAGGGCTCCTG
Sqle	ATAAGAAATGCGGGGATGTCAC	ATATCCGAGAAGGCAGCGAAC
Fdft1	ATGGAGTTCGTCAAGTGTCTAGG	CGTGCCGTATGTCCCCATC
Tm7sf2	AGCTTCGGGGGAGTCCTTC	CCAGGCACGGCCATACTTTT

### Western blotting

Total protein was extracted from mouse lung tissue and macrophages using RIPA buffer containing (P0013B, Beyotime Biotechnology, China) protease inhibitors. The protein concentration was determined with a BCA kit (Shanghai Epizyme Biomedical Technology, China), followed by separation of the proteins on a 10% SDS‒PAGE gel (Shanghai Epizyme Biomedical Technology). After SDS‒PAGE, the protein bands were transferred onto nitrocellulose membranes and blocked with Tris-buffered saline containing 0.1% Tween 20 and 5% bovine serum albumin for 1 h. The membrane was subsequently incubated overnight at 4 °C with primary antibodies (ratio: 1:1000) against κ-OR (57,282, Signalway Antibody, USA), stearoyl coenzyme desaturase 1 (SCD1, 2794S, Cell Signaling Technology, USA), NOD-like receptor protein 3 (NLRP3, 15,101, Cell Signaling Technology), IL-1β (ab283818, Abcam, USA), TNF-α (44,074, Signalway Antibody), IL-6 (ab229381, Abcam), smooth muscle 22 alpha (SM22α, Cat No.60213-1-Ig, Proteintech), and β-actin (Cat No.66009-1-Ig, Proteintech). Horseradish peroxidase-labeled goat anti-mouse IgG (A0216, Beyotime Biotechnology, China) and goat anti-rabbit IgG (A0208, Beyotime Biotechnology) were then applied at 37 °C for 1 h at a dilution ratio of 1:5000. Images were obtained using enhanced chemiluminescent-plus reagent (Biosharp Life Sciences, China) and analyzed with ImageJ.

### Statistical analysis

The data were presented as mean ± standard errors of the mean. Comparisons of only two groups were performed via the student's *t*-test (two-tailed). One-way ANOVA followed by the Bonferroni post hoc correction was used to compare differences among three or more groups. All the statistical analyses were performed with GraphPad Prism 8.4 (La Jolla, USA). The results were considered statistically significant when *p* < 0.05.

## Results

### Macrophage-specific κ-OR deficiency aggravated the progression of chronic HPH and the pulmonary inflammatory response

**Figure 1:**
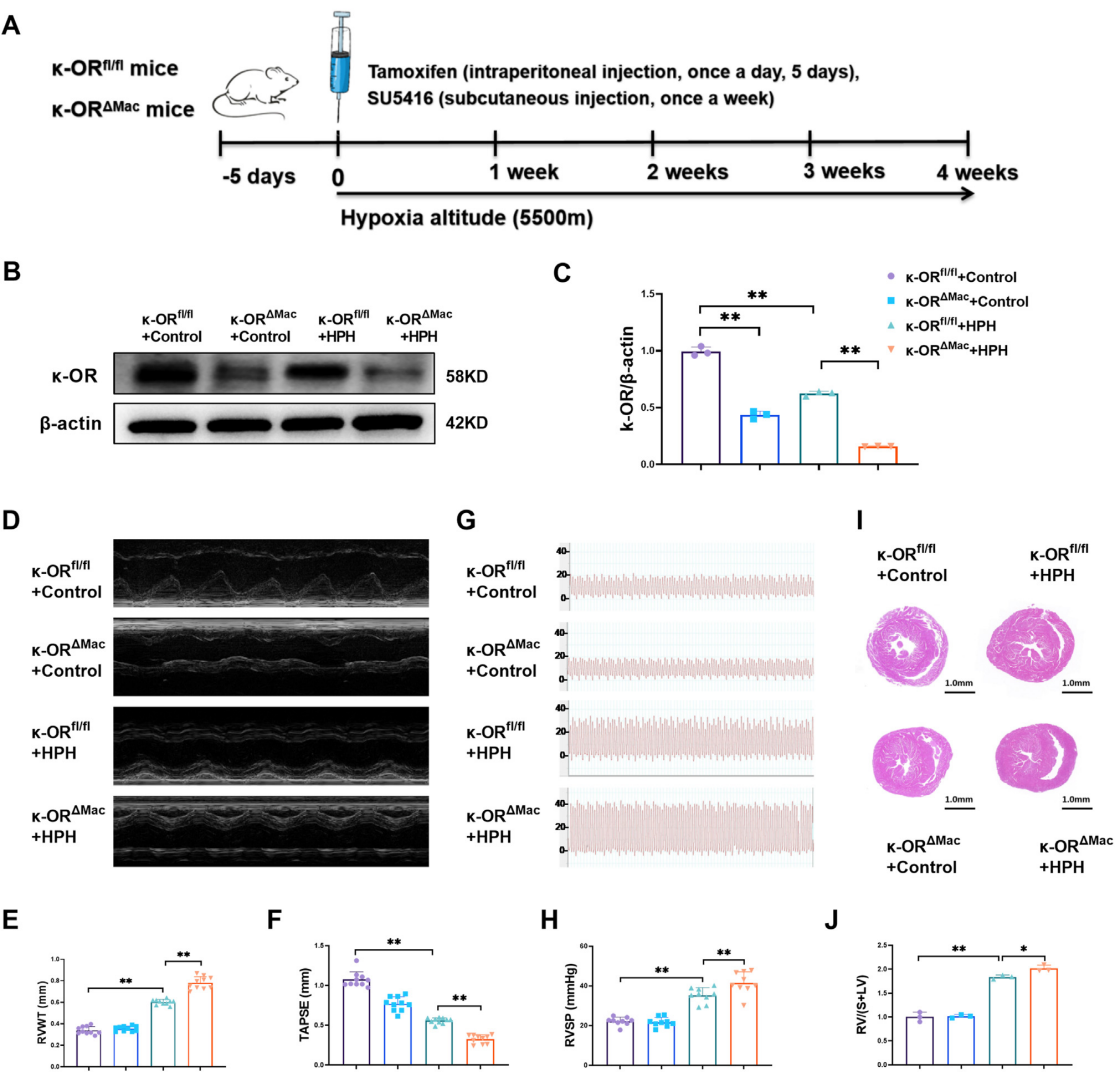
Macrophage-specific κ-OR deficiency aggravated chronic hypoxia-induced pulmonary hypertension (HPH). **(A)** Flow chart of the animal experiments. After 5 days of tamoxifen administration, κ-OR^fl/fl^ and κ-OR^ΔMac^ mice were exposed to indoor air (normoxia) or 10 % oxygen (hypoxia) plus weekly subcutaneous injection of SU5416 (10 mg/kg) for 4 weeks. **(B, C)** Mouse peritoneal macrophages were isolated 4 weeks after HPH, and κ-OR protein expression was analyzed by western blotting (*n* = 3 animals). **(D**–**F)** Echocardiography revealed changes in right ventricular wall thickness (RVWT) and tricuspid ring systolic displacement (TAPSE) values (*n* = 10 animals/group). **(G, H)** Changes in right ventricular systolic pressure were determined using a micro-pressure sensor (*n* = 9 animals/group). **(I, J)** Changes in the right heart hypertrophy index (*n* = 3 slices). ∗*p* < 0.05 and ∗∗*p* < 0.01. κ-OR^fl/fl^, mice with wild-type κ-OR in myeloid cells. κ-OR^ΔMac^, mice with myeloid-specific deletion of κ-OR.

Both κ-OR^fl/fl^ and κ-OR^ΔMac^ mice were subjected to a hypobaric hypoxia simulation chamber plus subcutaneous injection of SU5416 for 4 weeks to establish HPH models (Fig. 1A). κ-OR protein expression in mouse peritoneal macrophages was significantly lower in the κ-OR^fl/fl^ + HPH group than in the κ-OR^fl/fl^ + Control group (Fig. 1B, C). The echocardiography results (Fig. 1D–F) demonstrated that HPH led to increased right ventricular wall thickness and decreased tricuspid annular plane systolic excursion in κ-OR^fl/fl^ mice. We collected right heart hemodynamic data, which revealed a significant increase in right ventricular systolic pressure in κ-OR^ΔMac^ + HPH mice (Fig. 1G, H). The data from the histological heart sections revealed significantly increased right ventricular hypertrophy in the κ-OR^ΔMac^ + HPH mice (Fig. 1I, J).

Compared with κ-OR^fl/fl^ + Control mice, κ-OR protein expression in peritoneal macrophages isolated from κ-OR^ΔMac^ + Control mice was significantly lower (Fig. 1B, C). κ-OR knockdown in macrophages further increased right ventricular wall thickness and reduced tricuspid annular plane systolic excursion, as well as impaired right heart function (Fig. 1D–F) in HPH group mice. Furthermore, hemodynamic and histological studies revealed significantly greater right ventricular systolic pressure and right heart hypertrophy in the κ-OR^ΔMac^ + HPH group than in the κ-OR^fl/fl^ + HPH group (Fig. 1G–J). Collectively, these data suggest that macrophage κ-OR inhibits HPH progression and right heart dysfunction.

**Figure 2:**
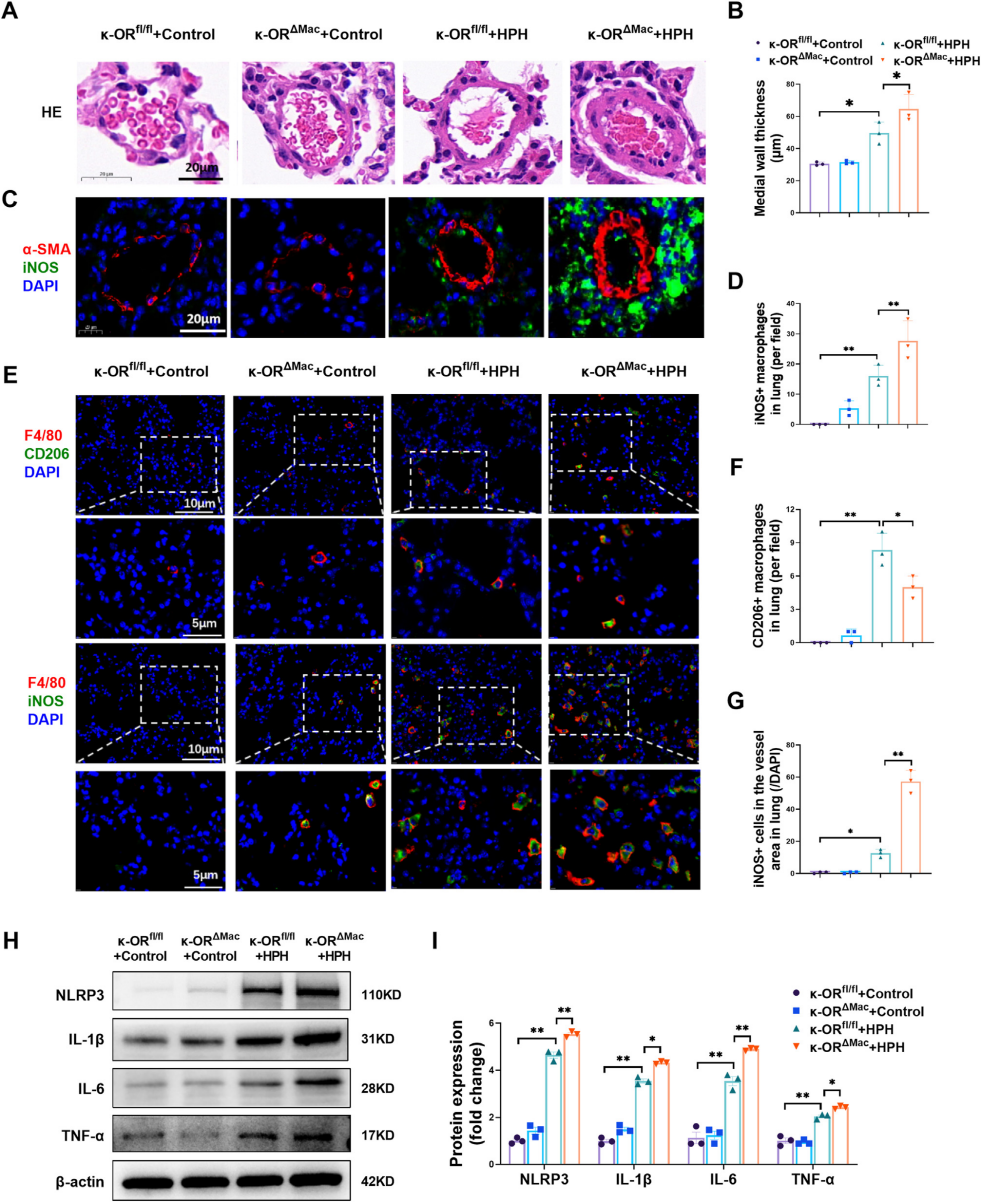
Macrophage-specific κ-OR deficiency aggravated the progression of chronic hypoxia-induced pulmonary vascular remodeling and the pulmonary inflammatory response. **(A, B)** Hematoxylin-eosin staining of mouse lung sections (*n* = 3 animals/group; scale bar, 20 μm). **(C, D)** Double staining of α-SMA (red) and iNOS (green) in the perivascular area in mouse lung sections (*n* = 3 animals/group; scale bar, 20 μm). **(E**–**G)** Immunostaining and quantification of iNOS (green) and F4/80 (red) double-positive cells and CD206 (green) and F4/80 (red) double-positive cells in mouse lung sections (*n* = 3 animals/group; scale bars, 10 or 5 μm). **(H, I)** NLRP3, IL-1β, IL-6, and TNF-α protein expression in the lungs of the four groups of mice was analyzed by western blotting (*n* = 3 animals/group). ∗*p* < 0.05 and ∗∗*p* < 0.01. κ-OR^fl/fl^, mice with wild-type κ-OR in myeloid cells. κ-OR^ΔMac^, mice with myeloid-specific deletion of κ-OR.

We further performed hematoxylin-eosin and immunofluorescence staining of lung sections, which revealed significant remodeling of small blood vessels within the distal pulmonary artery in κ-OR^fl/fl^ + HPH mice (Fig. 2A–D). Knockdown of macrophage κ-OR exacerbated PAMSC proliferation and vascular remodeling after HPH (Fig. 2C, D). In κ-OR^fl/fl^ + HPH mice, we found significantly increased infiltration of F4/80- and iNOS-positive macrophages in the lung tissue (Fig. 2E–G). Interestingly, knockdown of macrophage κ-OR significantly decreased the number of CD206-positive macrophages but increased the number of iNOS-positive macrophages (Fig. 2E–G). Western blotting revealed increased protein expression of inflammatory cytokines (IL-1β, IL-6, and TNF-α) and NLRP3 in the lung tissue of κ-OR^fl/fl^ + HPH mice (Fig. 2H, I). Knockdown of macrophage κ-OR further increased the protein expression of NLRP3 and these inflammatory cytokines (Fig. 2H, I). Overall, these results demonstrated that the knockdown of macrophage κ-OR not only aggravated hypoxia-induced M1 macrophage infiltration and perivascular inflammation but also worsened pulmonary artery remodeling and cardiac dysfunction.

### Macrophage-specific κ-OR deficiency exacerbated the lipopolysaccharide (LPS)-induced inflammatory response

**Figure 3:**
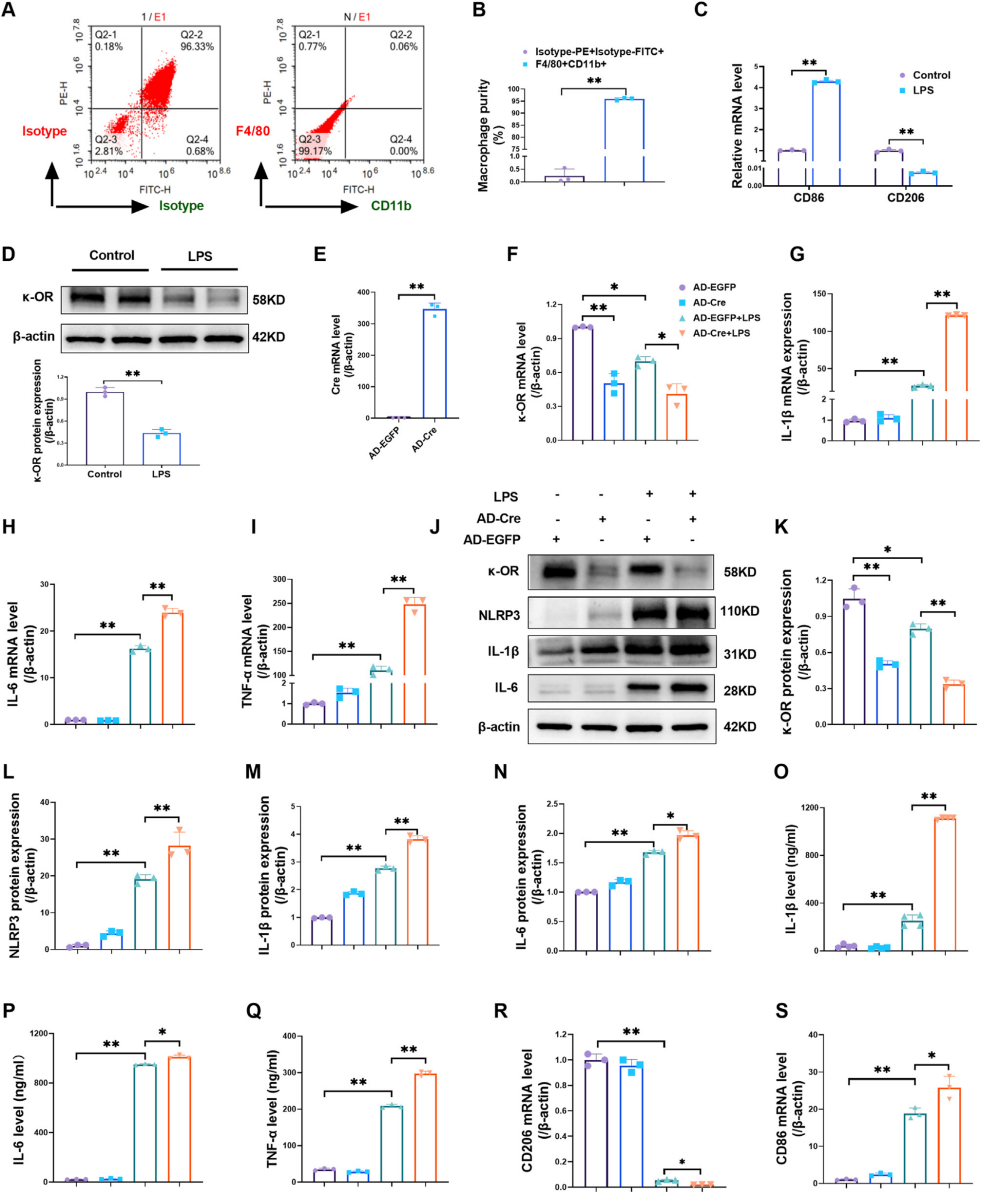
Macrophage-specific down-regulation of κ-OR aggravated the LPS-induced inflammatory response. **(A, B)** Peritoneal macrophages were isolated from mice 3 days after the administration of thioglycolate. The purity of the macrophages was assessed using flow cytometry for F4/80 and CD11b (*n* = 3). **(C)** The mRNA levels of CD86 and CD206 in macrophages were analyzed by quantitative real-time PCR (*n* = 3). **(D)** Western blotting and quantification of κ-OR protein expression in mouse peritoneal macrophages (*n* = 3). **(E)** Cre mRNA levels were analyzed using quantitative real-time PCR in macrophages infected with AD-EGFP or AD-Cre (*n* = 3). **(F–I)** Quantitative real-time PCR analysis of κ-OR, IL-1β, IL-6, and TNF-α mRNA expression in macrophages infected with AD-EGFP or AD-Cre followed by LPS treatment (*n* = 3). **(J**–**N)** Representative western blots and quantification of κ-OR, NLRP3, IL-1β, and IL-6 protein expression in mouse peritoneal macrophages (*n* = 3). **(O**–**Q)** IL-1β, IL-6, and TNF-α levels in the culture medium of mouse peritoneal macrophages were detected via ELISA (*n* = 3). **(R, S)** CD206 and CD86 mRNA expression was analyzed using quantitative real-time PCR (*n* = 3). ∗*p* < 0.05 and ∗∗*p* < 0.01. LPS, lipopolysaccharide, 100 ng/mL, 24 h; AD-EGFP, adenovirus carrying EGFP, 48 h; AD-Cre, adenovirus carrying EGFP and Cre recombinase, 48 h.

Mouse peritoneal macrophages were isolated for *in vitro* experiments. The peritoneal macrophages were assessed using flow cytometry for F4/80 and CD11b, demonstrating greater than 95 % of purity (Fig. 3A, B). We treated peritoneal macrophages with LPS and found that LPS significantly increased CD86 mRNA expression but decreased CD206 mRNA expression (Fig. 3C). Consistent with the *in vivo* data, LPS significantly decreased κ-OR protein expression in macrophages (Fig. 3D). We then established *in vitro* κ-OR-deficient peritoneal macrophages via infection with adenovirus carrying EGFP and Cre recombinase (AD-Cre) (Fig. 3E). Control macrophages were infected with adenovirus carrying only EGFP (AD-EGFP) (Fig. 3E). Macrophages infected with AD-EGFP or AD-Cre were treated with or without LPS for 24 h. Then, κ-OR and inflammatory cytokine mRNA and protein expression were assessed (Fig. 3F–Q). AD-Cre significantly reduced κ-OR mRNA (Fig. 3F) and protein (Fig. 3J, K) expression in macrophages, both in the presence and absence of LPS. Furthermore, macrophage κ-OR knockdown significantly increased IL-1β, IL-6, and TNF-α mRNA expression (Fig. 3G–I); NLRP3, IL-1β, and IL-6 protein expression (Fig. 3J, L–N); and the release of IL-1β, IL-6, and TNF-α upon LPS stimulation (Fig. 3O–Q). Additionally, peritoneal macrophages with κ-OR deficiency exhibited polarization toward the M1 but not the M2 phenotype, as evidenced by increased CD86 and decreased CD206 mRNA levels (Fig. 3R, S). These findings suggest that κ-OR exerts an anti-inflammatory effect on macrophages, which is consistent with the *in vivo* data from mice in the κ-OR^ΔMac^ + HPH group.

### Activating κ-OR with U50,488H inhibited the inflammatory response and paracrine function of macrophages

**Figure 4:**
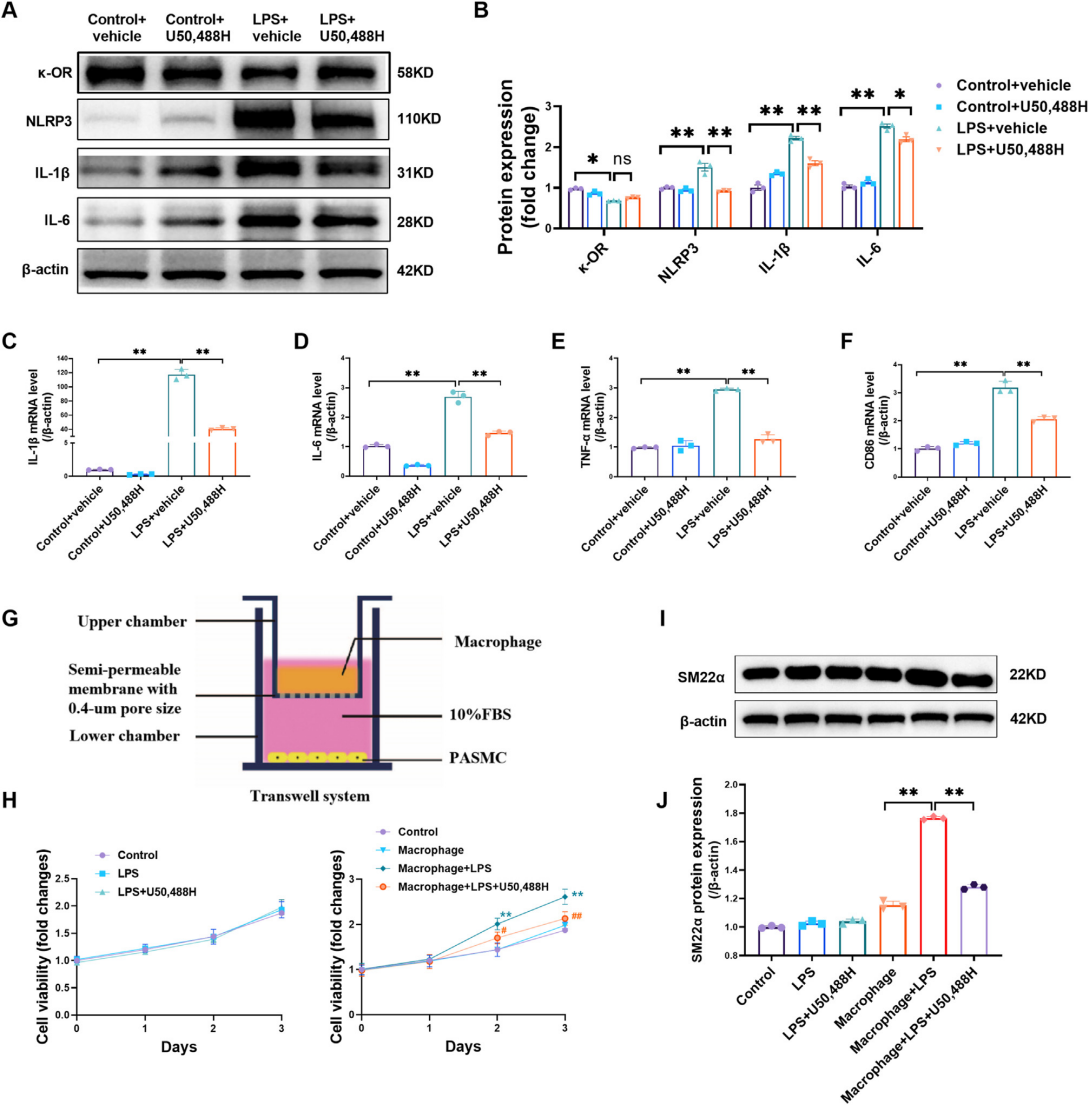
Activating κ-OR with U50,488H inhibited the inflammatory response and paracrine function of macrophages. **(A, B)** Western blotting and quantification of κ-OR, NLRP3, IL-1β, and IL-6 protein expression in mouse peritoneal macrophages (*n* = 3). **(C–F)** Quantitative real-time PCR was used to detect IL-1β, IL-6, TNF-α, and CD86 mRNA expression in mouse peritoneal macrophages (*n* = 3). **(G)** Transwell coculture diagram for macrophages (upper chamber) and PASMCs (lower chamber). **(H)** Cell viability assays with a CCK-8 kit showed the effects of LPS, U50,488H, and/or macrophages on PASMC proliferation over 3 days (*n = 3*). **(I)** Western blotting and quantification of SM22α protein expression in PASMCs after coculture with macrophages in the presence or absence of LPS, U50,488H (*n* = 3). ∗*p* < 0.05, ∗∗*p* < 0.01. PASMC, pulmonary artery smooth muscle cell; LPS, lipopolysaccharide, 100 ng/mL, 24 h; U50,488, 40 μM, 24 h.

Given that κ-OR knockdown in macrophages aggravates the inflammatory response of macrophages, we next investigated the role of the κ-OR selective agonist U50,488H in LPS-induced macrophage inflammation. Peritoneal macrophages were treated with 40 μM U50,488H 10 min before 100 ng/mL LPS administration. U50,488H did not significantly alter κ-OR protein expression in macrophages (Fig. 4A, B). However, U50,488H significantly reduced the LPS-induced macrophage inflammatory response, as evidenced by decreased NLRP3, IL-1β, and IL-6 protein expression (Fig. 4A, B) and decreased IL-1β, IL-6, TNF-α, and CD86 mRNA expression (Fig. 4C–F). These results indicate that the activation of κ-OR exerts anti-inflammatory effects on macrophages.

To further explore the potential effect of macrophage κ-OR activation on PASMC proliferation, a transwell coculture experiment was performed (Fig. 4G). In the absence of macrophages, PASMC proliferation and SM22α protein expression did not significantly change after LPS or LPS plus U50,488H administration (Fig. 4H–J). However, in the presence of macrophages, LPS significantly increased PASMC proliferation and SM22α protein expression, which was almost completely blocked by U50,488H (Fig. 4H–J). These data suggest that κ-OR activation in macrophages exerts anti-proliferative effects on PASMCs via a paracrine mechanism.

### κ-OR activation exerted anti-inflammatory effects on macrophages via the up-regulation of SCD1

**Figure 5:**
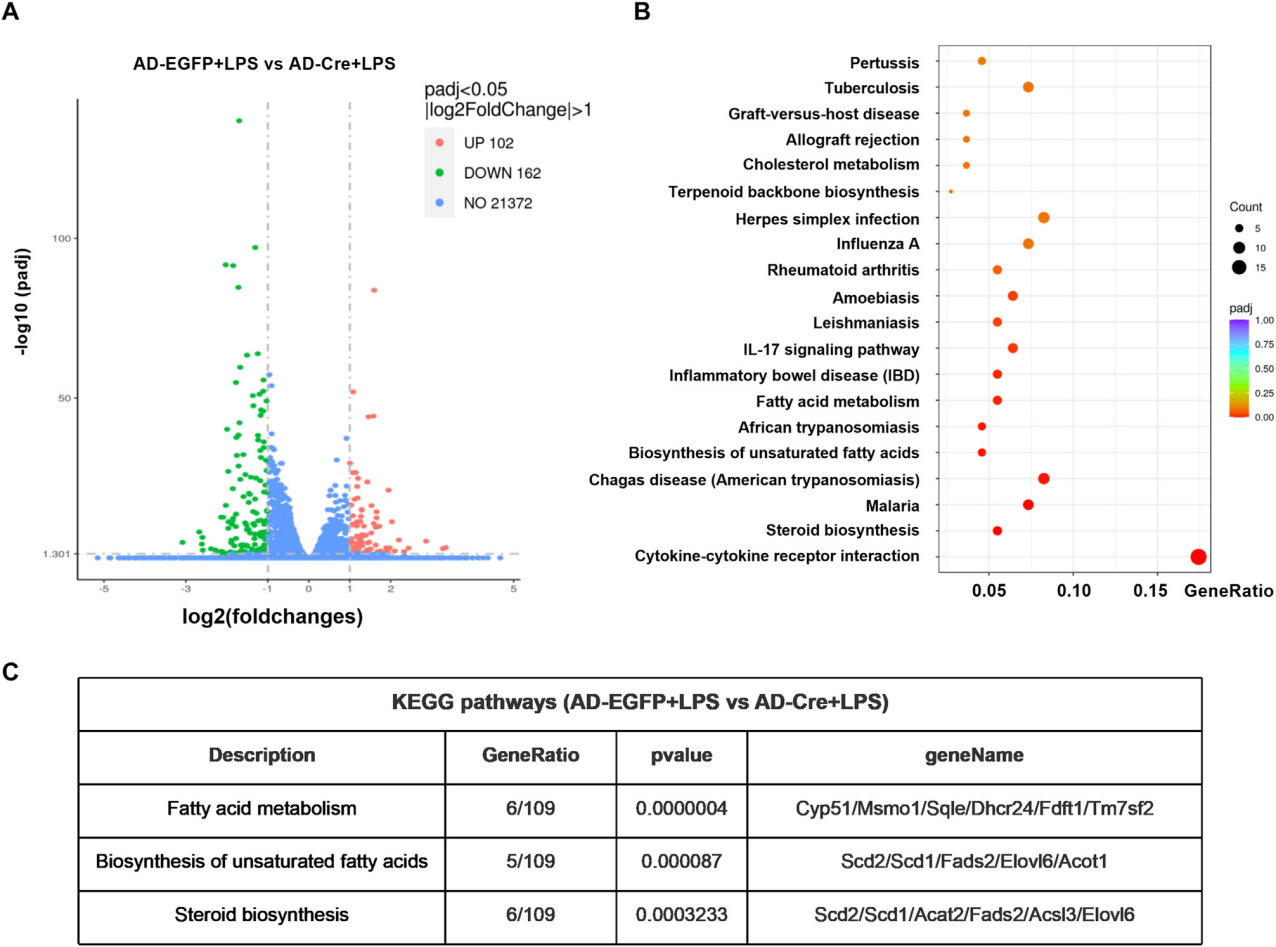
RNA sequencing data of peritoneal macrophages from the AD-EGFP + LPS and AD-Cre + LPS groups. **(A)** The volcano plot showed the differentially expressed genes in macrophages between the AD-EGFP + LPS and AD-Cre + LPS groups. The 102 up-regulated genes in the AD-EGFP + LPS group are represented by red dots, and the 162 down-regulated genes in the AD-EGFP + LPS group are represented by green dots. **(B)** The top 20 significantly changed KEGG pathways in peritoneal macrophages between the AD-EGFP + LPS and AD-Cre + LPS groups. **(C)** The corresponding genes in the KEGG pathways of steroid biosynthesis, unsaturated fatty acid biosynthesis, and fatty acid metabolism. LPS, lipopolysaccharide; AD-EGFP, adenovirus carrying EGFP; AD-Cre, adenovirus carrying EGFP and Cre recombinase.

We then performed RNA sequencing experiments to understand the mechanisms underlying κ-OR in macrophages from the Ad-Cre + LPS group and the Ad-EGFP + LPS group (Fig. 5A). A total of 264 significantly differentially expressed genes in macrophages were identified between the AD-EGFP + LPS and AD-Cre + LPS groups (Fig. 5A). KEGG pathway enrichment analysis revealed the involvement of various pathways related to the inflammatory response, such as cytokine–cytokine receptor interaction, malaria, Chagas disease, inflammatory bowel disease, the IL-17 signaling pathway, and leishmaniasis, among the top 10 differentially expressed pathways (Fig. 5B), which supports the important role of κ‒OR in the macrophage-mediated anti-inflammatory response. Interestingly, in addition to inflammation-related pathways, many lipid metabolism-related pathways, such as steroid biosynthesis, biosynthesis of unsaturated fatty acids, and fatty acid metabolism, were enriched in the KEGG pathway analysis among the top 10 differentially expressed pathways.

**Figure 6:**
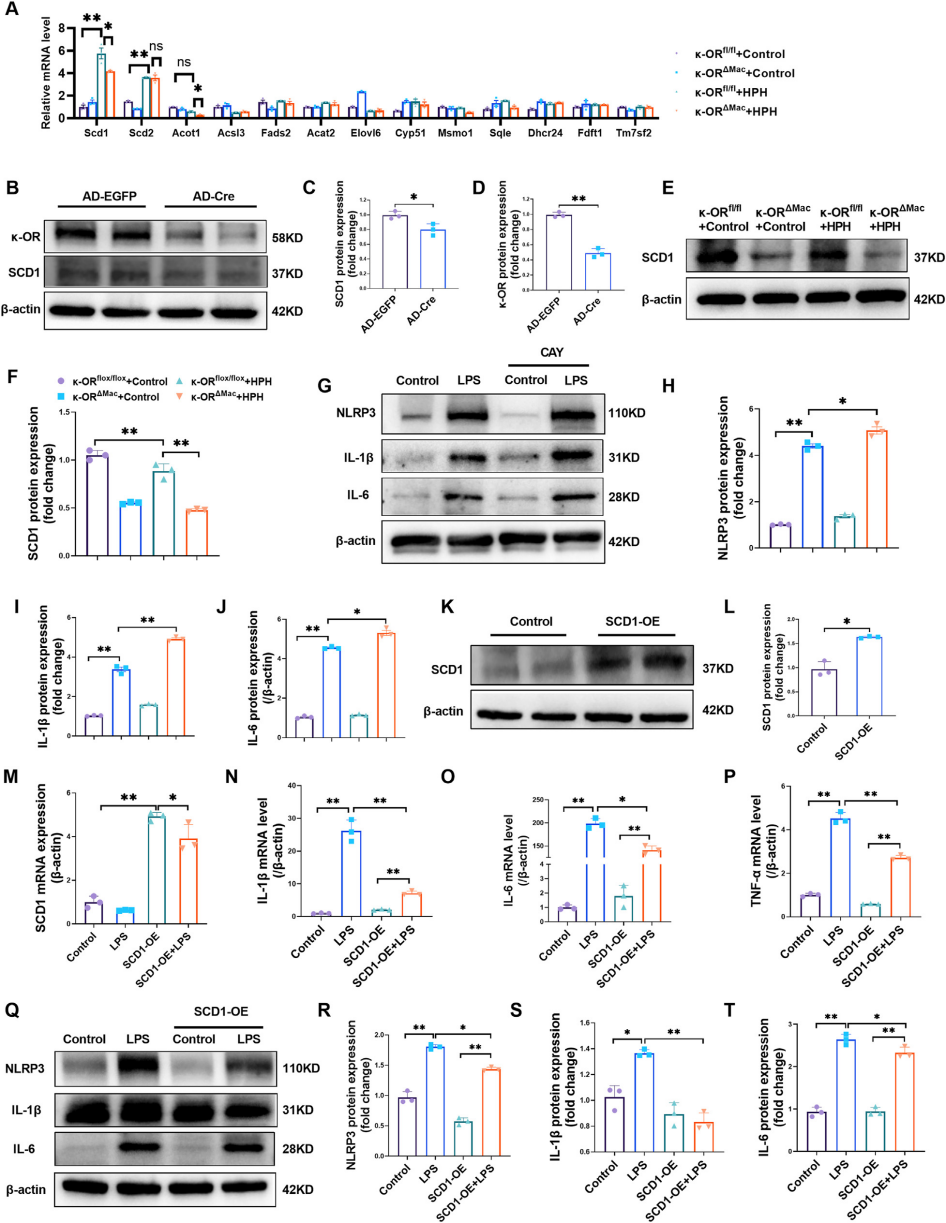
κ-OR activation exerted an anti-inflammatory effect on macrophages via the up-regulation of SCD1. **(A)** Scd1, Scd2, Acot1, Acsl3, Fads2, Acat2, Elovl6, Cyp51, Msmo1, Sqle, Dhcr24, Fdft1, and Tm7sf2 mRNA expression in mouse peritoneal macrophages (*n* = 3). **(B**–**D)** Western blotting and quantification of κ-OR and SCD1 protein expression in mouse peritoneal macrophages (*n* = 3). **(E, F)** Western blotting and quantification of SCD1 in mouse peritoneal macrophages (*n* = 3). **(G**–**J)** Western blotting and quantification of NLRP3, IL-1β, and IL-6 in macrophages (*n* = 3). **(K, L)** Western blotting and quantification of SCD1 in RAW264.7 cells 48 h after infection with a lentivirus harboring SCD1 (SCD1-OE) (*n* = 3). **(M**–**P)** mRNA expression of SCD1, IL-1β, IL-6, and TNF-α in RAW264.7 cells treated with LPS (*n* = 3). **(Q**–**T)** Western blotting and quantification of NLRP3, IL-1β, and IL-6 in lung tissue (*n* = 3). ∗*p* < 0.05 and ∗∗*p* < 0.01. LPS, lipopolysaccharide, 100 ng/mL, 24 h; AD-EGFP, adenovirus carrying EGFP, 48 h; AD-Cre, adenovirus carrying EGFP and Cre recombinase, 48 h. CAY, CAY10566, 7.5 μM.

Accumulating evidence has shown that metabolic dysfunction, especially abnormal fatty acid metabolism, plays an important role in the pathophysiology of PH.^31^^,^^32^ We then performed quantitative real-time PCR to test the genes involved in steroid biosynthesis, unsaturated fatty acid biosynthesis, and fatty acid metabolism (Fig. 5C). Among these 13 genes, SCD1 was significantly increased in the peritoneal macrophages of the κ-OR^fl/fl^ + HPH group but was decreased in those of the κ-OR^ΔMac^ + HPH group (Fig. 6A). SCD is recognized as a pivotal regulator of lipid homeostasis and is implicated in metabolic syndrome development and inflammation.^33^ SCD1 serves as a rate-limiting enzyme to produce monounsaturated fatty acids involved in triglyceride synthesis and the composition of membrane phospholipids. Macrophage-specific SCD1 has been linked to metabolic syndrome progression and associated inflammation.^34^ Therefore, investigating the relationship between macrophage-specific SCD1 expression levels and the inflammatory response is highly important.

Western blotting analysis further revealed that κ-OR deficiency resulted in a significant decrease in SCD1 protein expression in isolated peritoneal macrophages (Fig. 6B–D). We then tested SCD1 protein expression in the lung tissue of mice in the κ-OR^fl/fl^ + Control, κ-OR^ΔMac^ + Control, κ-OR^fl/fl^ + HPH, and κ-OR^ΔMac^ + HPH groups. Both κ-OR deficiency and HPH significantly down-regulated SCD1 protein expression in lung tissues (Fig. 6E, F). We then treated macrophages with CAY10566 (CAY), a chemical inhibitor of SCD1,^35^ which significantly increased the LPS-induced NLRP3, IL-1β, and IL-6 protein expression (Fig. 6G–J). We also infected macrophages with a lentivirus harboring SCD1 (Fig. 6K–M). Lentivirus-mediated SCD1 overexpression significantly decreased IL-1β, IL-6, and TNF-α mRNA expression levels following LPS stimulation in macrophages (Fig. 6N–P). Moreover, SCD1 overexpression significantly suppressed NLRP3, IL-1β, and IL-6 protein expression in LPS-treated macrophages (Fig. 6Q–T). Overall, these data revealed an inhibitory effect of SCD1 on the macrophage inflammatory response. These findings suggest that increasing SCD1 expression may inhibit HPH by attenuating the release of inflammatory cytokines.

## Discussion

HPH is a chronic progressive cardiovascular disease that poses a significant threat to human health; it is triggered primarily by continuous hypoxic stimulation.^11^ HPH is strongly associated with right heart failure and mortality in patients.^36^ HPH is characterized by pulmonary vasoconstriction and pulmonary vascular remodeling. Chronic hypoxia induces various changes, including endothelial damage, smooth muscle proliferation, abnormal gene expression, cytokine and growth factor imbalance, and the activation of ion channels and kinases,^37^, ^38^, ^39^ leading to the development of HPH. To date, the underlying mechanism of pulmonary vascular remodeling is not well understood.

In the present study, we report several novel findings. First, we found that κ-OR expression in macrophages was significantly decreased after HPH and that macrophage-specific κ-OR knockdown exacerbated HPH. In 2009, our team detected κ-OR expression in PASMCs via immunohistochemistry and reported the expression of κ-OR in the pulmonary artery for the first time.^40^ In the present study, we isolated peritoneal macrophages and observed high κ-OR expression in macrophages, which was significantly decreased after HPH. κ-OR represents a major type of opioid receptor within the cardiovascular system that can be selectively activated by U50,488H administration. Our previous studies have demonstrated that κ-OR activation induced by U50,488H plays a pivotal role in attenuating HPH.^41^^,^^42^ Moreover, κ-OR activation inhibits the expression of inflammatory cytokines and adhesion factors in pulmonary artery endothelial cells and cardiomyocytes induced by LPS, revealing κ-OR-mediated immune regulation.^26^^,^^27^^,^^43^ However, the potential role of macrophage κ-OR during the development of HPH remains unclear. Therefore, we constructed macrophage-specific κ-OR knockout mice (κ-OR^ΔMac^) and reported that κ-OR deficiency in macrophages significantly aggravated pulmonary vascular remodeling and right heart hypertrophy and decreased cardiac function, aggravating the course of HPH in these mice. These results demonstrated that macrophage κ-OR inhibits HPH progression and right heart dysfunction.

Second, we found that macrophage κ-OR inhibited HPH by inhibiting inflammatory responses. Perivascular inflammatory infiltration is a significant pathological feature of most PH patients. Previous studies have shown that during the pathological progression of PH, a variety of immune cells, including neutrophils and macrophages, accumulate around pulmonary vessels. Moreover, high levels of cytokines, chemokines, and inflammatory factors have been detected in PH patients,^44^^,^^45^ indicating that inflammation plays a very important role in PH and pulmonary vascular remodeling. Our studies, including the present one, have shown that κ-OR activation has a clear anti-HPH effect; however, whether the mechanism is related to the regulation of immune function is unclear.^25^^,^^26^^,^^28^ Therefore, we conducted *in vitro* and *in vivo* studies. *In vivo*, a mouse model of HPH was successfully established via hypoxia and subcutaneous injection of SU5416 for 4 weeks. The results revealed a significant increase in right ventricular pressure and the right ventricular hypertrophy index, whereas the right ventricular pressure and right ventricular hypertrophy index were further increased in κ-OR^ΔMac^ + HPH mice after 4 weeks of hypoxia compared with those in κ-OR^fl/fl^ + HPH mice. Echocardiographic results revealed that compared with κ-OR^fl/fl^ + HPH mice, κ-OR^ΔMac^ + HPH mice presented further reduced cardiac function. Immunofluorescence and hematoxylin-eosin staining revealed that the small vessels in the distal pulmonary arteries of κ-OR^fl/fl^ + HPH mice were significantly remodeled and had increased infiltration of macrophages around the pulmonary vessels. These vessels contained both M1-type (iNOS) and M2-type (CD206) phenotypes of macrophages, and M1-type macrophages were predominant, whereas κ-OR^ΔMac^ + HPH mice presented further aggravated vascular remodeling and increased infiltration of M1-type macrophages. Western blotting experiments revealed that compared with κ-OR^fl/fl^ + HPH mice, κ-OR^ΔMac^ + HPH mice expressed higher levels of NLRP3 and the inflammatory cytokines IL-6, IL-1β, and other proteins in their lung tissue. *In vitro*, compared with LPS-treated control macrophages, LPS-treated macrophages with κ-OR knockdown presented significantly increased expression of inflammatory cytokines and obvious M1-type polarization. This finding is consistent with the observation of infiltrated M1 macrophages around pulmonary vessels after hypoxic treatment. In the LPS-treated group, if the κ-OR selective agonist U50,488H was given in advance, the macrophage inflammatory response was inhibited. The coculture of macrophages and PASMCs revealed that U50,488H inhibited the macrophage inflammatory response and inhibited the proliferation of PASMCs. These results suggest that κ-OR exerts anti-inflammatory effects to inhibit HPH progression.

Finally, we revealed that SCD1 mediates κ-OR-induced anti-inflammatory responses in macrophages. In addition to pulmonary vascular resistance and right ventricular hypertrophy, metabolic dysfunction also plays a major role in the pathophysiology of PH. Inhibition of fatty acid synthase can reduce pulmonary vascular remodeling, right ventricular pressure, and right ventricular hypertrophy and improve endothelial function, thereby reducing PH, indicating that fatty acid metabolism is also involved in the process of PH.^31^ SCD1 is an enzyme that catalyzes the generation of monounsaturated fatty acid-major triglyceride components stored in lipid droplets from saturated fatty acid substrates.^46^ A recent study reported that exercise-induced increases in SCD1 expression mitigate NF-κB-mediated inflammatory responses in vessels.^47^ Reduced SCD1 expression in macrophages leads to a change in membrane lipid composition and an increase in STAT1 tyrosine phosphorylation driven by TLR4, which ultimately leads to an increase in proinflammatory cytokine secretion.^34^ In the present study, we found that the down-regulation of κ-OR in macrophages caused a decrease in SCD1 expression. Pharmacological inhibition of SCD1 with CAY10566 significantly increased LPS-induced NLRP3, IL-1β, and IL-6 expression. SCD1 overexpression significantly inhibited the LPS-induced inflammatory response in macrophages.

As a member of the opioid receptor family, κ-OR is expressed in the central and peripheral nervous systems as well as on the surface of different types of immune cells, *e.g.*, T cells, B cells, monocytes, and macrophages. Macrophages play the most important role in inflammation and tissue damage, including the removal of cell debris or activation, which leads to the resolution of inflammation. In the present study, we observed decreased κ-OR expression in macrophages in an HPH mouse model. Knockdown of κ-OR in macrophages not only aggravated hypoxia-induced pulmonary vascular remodeling and right ventricular hypertrophy and decreased cardiac function but also aggravated the infiltration and inflammatory response of M1 macrophages around pulmonary vessels, which aggravated PASMC proliferation and HPH progression. The selective agonist U50,488H of κ-OR significantly inhibited the inflammatory response of macrophages, thereby inhibiting PASMC proliferation and effectively alleviating the process of HPH. We further described the interaction between κ-OR and SCD1 in macrophages. Down-regulation of κ-OR expression in macrophages leads to a decrease in SCD1 expression, whereas SCD1 overexpression improves the inflammatory response and provides a potential treatment for HPH.

Selected κ-OR receptor ligands, such as U50,488H and salvinorin A, have been characterized in preclinical and clinical studies.^48^ We and others have reported that U50,488H exerts anti-inflammatory effects in several diseases, such as myocardial ischemia/reperfusion and osteoarthritis synovitis.^27^^,^^49^ Here, we describe a new cellular and molecular mechanism underlying the protective potential of macrophage κ-OR activation in HPH mice. A very recent study reported that salvinorin A protected against epileptic seizures and neuronal damage in pilocarpine-induced models by suppressing the inflammatory response through regulating microglial M1/M2 polarization.^50^ Therefore, our findings may also apply to other chronic inflammatory diseases where κ-ORs play important roles.

## CRediT authorship contribution statement

**Qiaojuan Wang:** Writing – original draft, Methodology, Investigation, Data curation, Conceptualization. **Jiayuan Liu:** Data curation. **Renqi Li:** Methodology. **Sihan Kong:** Methodology. **Yinjie Wang:** Validation. **Guoyang Huang:** Methodology, Conceptualization. **Shumiao Zhang:** Investigation, Conceptualization. **Na Feng:** Investigation. **Xiaoming Gu:** Methodology. **Yali Liu:** Methodology. **Ming Jia:** Investigation. **Feng Fu:** Investigation. **Jun Li:** Methodology. **Juan Li:** Project administration. **Jianming Pei:** Resources.

## Data availability

All the data of this study are free to obtain in the paper or the supplementary materials. The raw data that support the findings of this study are available from the corresponding author upon reasonable request.

## Funding

This work was financially supported by the Program for National Science Funds of China (No. 82070051), the Natural Science Basic Research Key Project of Shaanxi Province, China (No. 2022JZ-55), the National Defense Foundation Strengthening Project of the Science and Technology Commission (China) (No. JSLY-27-B27004), the Air Force Military Medical University Cross-Integration Project (China) (No. 2023JC017), the Military Medical Advancement Program of Air Force Military Medical University (China) (No. 2021JSTS11), and the 10.13039/501100015401Key Research and Development Projects of Shaanxi Province, China (No. 2023-YBSF-373).

## Conflict of interests

Feng Fu is one of the Editorial Board members of *Genes & Diseases*, but he/she has no involvement in the peer-review of this article and has no access to information regarding its peer-review. The authors declared no other competing interests.
